# Antimicrobial Studies on Extracts of Four Species of *Stachys*

**DOI:** 10.4103/0250-474X.43021

**Published:** 2008

**Authors:** M. Saeedi, K. Morteza-Semnani, M. R. Mahdavi, F. Rahimi

**Affiliations:** Department of Pharmaceutics, Faculty of Pharmacy, Mazandaran University of Medical Sciences, POBox 48175-861, Sari, Iran; 1 Department of Medicinal Chemistry, Faculty of Pharmacy, Mazandaran University of Medical Sciences, POBox 48175-86, Sari, Iran; 2 Department of Laboratory Sciences, Faculty of Paraclinical Sciences, Mazandaran Uniersity of Medical Sciences, POBox 48175-861, Sari, Iran

**Keywords:** Antimicrobial, extract, *Stachys*, MIC

## Abstract

The antimicrobial activity of the methanol extracts of the dried flowering aerial parts of *Stachys byzantina*, *S. inflata*, *S. lavandulifolia* and *S. laxa* (Labiatae) were studied using the disc diffusion method and determination of minimum inhibitory concentration (MIC) values against *Staphylococcus aureus*, *Streptococcus sanguis*, *Escherichia coli*, *Pseudomonas aeroginosa*, *Klebsiella pneumoniae*, *Aspergilus niger* and *Candida albicans*. The extracts of plants exhibited concentration-dependent antibacterial activity against the bacteria tested. The extracts were more active against Gram-positive microorganisms. The extracts, however, did not show any antifungal activity.

The sub cosmopolitan genus *Stachys* compromises more than 270 species and is justifiably considered as one of the largest genera of the Labiatae^1^. The genus *Stachys* includes 34 species in Iran^2^. Phytochemical investigations of *Stachys* species have shown the occurrence of flavonoids, diterpenes, phenyl ethanoid glycosides and saponins^3^. Plants of this genus have long been applied to treat genital tumors, sclerosis of the spleen, inflammatory tumors and cancerous ulcers^4^. *S. byzantina* C. Koch. (syn. *S. lanata* Jacq.), *S. inflata* Benth., *S. lavandulifolia* Vahl and *S. laxa* Boiss. and Buhse are aromatic plants, which grow in Azerbaijan, Golestan, Khorasan, Mazandaran and Tehran provinces of Iran^5^. A bibliographical survey showed that there were no reports on the antimicrobial activity of these species. In continuation of studies of Iranian species of the Labiatae family, we have had occasion to investigate the antimicrobial activity of *S. byzantina*, *S. inflata*, *S. lavandulifolia* and *S. laxa*.

The flowering aerial parts of *S. byzantina*, *S. inflata, S. lavandulifolia* and *S. laxa* were collected in May 2004 from the suburb of Behshahr, Mazandaran province, North of Iran and identified at the Department of Botany, Research Center of Natural Resources of Mazandaran). Voucher specimens (herbarium No. 151, 152, 154 and 155) were deposited in the Herbarium of the Department of Botany, Research Center of Natural Resources of Mazandaran. Dried plant materials were ground to fine powder. One hundred grams of the each powders were extracted twice with methanol. The extacts were evaporated to dryness at 40°, and stored in 4°.

*Staphylococcus aureus* PTCC 1112, *Streptococcus sanguis* PTCC 1449, *Escherichia coli* PTCC 1330, *Pseudomonas aeroginosa* PTCC 1074, *Klebsiella pneumoniae* PTCC 1053, *Aspergilus niger* PTCC 5011 and *Candida albicans* PTCC 5027 were used for testing the antimicrobial activity. *In vitro* antimicrobial studies were carried out by the disc diffusion method and minimum inhibitory concentration (MIC) values were determined against test microorganisms^6^. In the disc diffusion method, extracts were dissolved in methanol and applied to a 6 mm diameter paper disc. The extracts were tested at 10, 50, 100, 250, 500, 750 and 1000 μg/disc. Inhibition zone diameters were measured after 24 h. Gentamicin (50 μg/disc), amikacin (3 μg/disc) and amphotericin B (100 μg/disc, Sigma) were used as positive controls. MICs were determined by the dilution method at concentrations of 10 μg/ml to 25 mg/ml of culture medium^6^. Gentamicin (2 mg/ml) and amphotericin B (100 μg/ml) were used as positive controls.

The yield of methanol extracts of *Stachys byzantina*, *S. inflata*, *S. lavandulifolia* and *S. laxa* was 14.1%, 14.3%, 10.1% and 10.6% w/w, respectively. Tables 1 and 2 gives a summary of the results of the antimicrobial effects and MICs of *Stachys* species investigated. The methanol extracts of the dried flowering aerial parts of *S. byzantina*, *S. inflata*, *S. lavandulifolia* and *S. laxa* exhibited concentration-dependent antibacterial activity against bacteria tested. The methanol extracts were more active against Gram-positive microorganisms (*Streptococcus sanguis* and *Staphylococcus aureus* ). The extracts, however, did not show antifungal activity.

**TABLE 1:** ANTIMICROBIAL ACTIVITY OF THE METHANOL EXTRACTS OF *STACHYS BYZANTINA*, *S. INFLATA*, *S. LAVANDULIFOLIA* AND S. LAXA*

Sample	Con. (μg/disc)	Diameter of zone of inhibition (mm)				
		
		*Bacteria*				
		
		*S. aureus* (G +)	*S. sanguis* (G +)	*E. coli* (G -)	*P. aeroginosa* (G -)	*K. pneumoniae* (G -)
*S. byzantina*	10	-	-	-	-	-
	50	-	8.7	-	-	-
	100	8.6	10.1	8.5	-	-
	250	9.6	11.7	9.5	-	8.8
	500	10.9	12.7	11.1	-	10.1
	750	12.2	13.8	13.3	8.8	11.3
	1000	13.4	15.0	14.5	10.2	13.4
*S. inflata*	10	-	-	-	-	-
	50	-	-	-	-	-
	100	-	-	-	-	-
	250	8.3	8.6	-	9.0	8.7
	500	9.4	9.8	8.8	10.2	9.5
	750	10.8	11.2	9.6	11.3	10.7
	1000	11.9	12.4	10.7	12.7	11.7
*S. lavandulifolia*	10	-	-	-	-	-
	50	-	8.6	-	-	-
	100	-	9.5	-	-	8.5
	250	-	10.9	8.5	8.6	9.3
	500	8.6	11.7	9.5	9.7	10.9
	750	9.5	13.5	10.6	10.9	11.8
	1000	11.6	15.3	12.1	12.4	12.9
*S. laxa*	10	-	-	-	-	-
	50	-	-	-	-	-
	100	8.6	-	-	-	-
	250	10.0	8.6	-	8.5	-
	500	11.5	10.0	-	9.8	8.5
	750	13.2	11.3	-	11.6	10.1
	1000	14.2	13.4	-	12.6	12.2
Gentamicin	50	37.3	24.0	31.6	31.0	28.0
Amikacin	3	24.9	19.9	23.8	15.8	18.0
Amphotericin B	100	-	-	-	-	-

* Zone of inhibition, including the diameter of the filter paper disc (6 mm); mean value of eight independent experiments; gentamicin, amikacin and amphotericin B were used as positive controls; - no inhibition.

**TABLE 2:** MINIMUM INHIBITORY CONCENTRATION (MIC) OF *STACHYS BYZANTINA*, *S. INFLATA*, *S. LAVANDULIFOLIA* AND S. LAXA*

Sample	MIC (mg/ml)				
	
	*Bacteria*				
	
	*S. aureus* (G +)	*S. sanguis (G +)*	*E. coli (G -)*	*P. aeroginosa (G -)*	*K. pneumoniae (G -)*
*S. byzantina*	10	10	25	40	40
*S. inflata*	10	10	40	40	40
*S. lavandulifolia*	40	10	25	25	25
*S. laxa*	10	25	-	40	40

* All determinations were done in triplicate; gentamicin (2 mg/ml) and amphotericin B (100 μg/ml) were used as positive controls

In 2004 and 2005, the antimicrobial activity of some endemic *Stachys* species including *S. sivasica*, *S. anamurensis*, *S. cydnia*, *S. aleurites* and *S. pinardii* was reported; the methanol extracts of *Stachys* L. were effective only against bacteria tested^7^,^8^. In 2005, essential oils and ethanol extracts from the leaves and/or roots of 35 medicinal plants commonly used in Brazil were screened for anti*Candida albicans* activity; essential oils from 13 plants including *S. byzantina* showed anti-*Candida* activity; the ethanol extract was not effective at any of the concentrations tested^9^.

In the present study, the results concluded that the methanol extracts of these plants have a potential as source of antibacterial agent of natural origin. Preliminary phytochemical studies showed that the aerial parts of the genus *Stachys* contain flavonoids. Flavonoids may be responsible for their antibacterial activity.
